# Long COVID-19 Enigma: Unmasking the Role of Distinctive Personality Profiles as Risk Factors

**DOI:** 10.3390/jcm13102886

**Published:** 2024-05-14

**Authors:** Dana Amsterdam, Aviv Kupershmidt, Asia Avinir, Ron Matalon, Ofir Ohana, Omri Feder, Shai Shtrozberg, Guy Choshen, Jacob Nadav Ablin, Odelia Elkana

**Affiliations:** 1 Department of Internal Medicine H, Tel Aviv Medical Center, 6 Weizmann St., Tel Aviv 6423906, Israel; avivku@tlvmc.gov.il (A.K.); omrife@tlvmc.gov.il (O.F.); shaisht@tlvmc.gov.il (S.S.); 2 School of Behavioral Sciences, The Academic College of Tel-Aviv—Yaffo, Tel Aviv 6818211, Israel; asiavinir@gmail.com (A.A.); elkana@mta.ac.il (O.E.); 3 Department of Internal Medicine B, Meir Medical Center, 59 Tsharnehovski St., Kfar Saba 4428163, Israel; meguyhos@clalit.org.il

**Keywords:** Long COVID, type D personality, personality traits, anxiety, depression, fibromyalgia, cognitive decline, sleep disturbances, quality of life

## Abstract

**Background:** The COVID-19 (Coronavirus disease 2019) pandemic has prompted extensive research into lingering effects, especially in ‘Long COVID’ patients. Despite exploration, contributing factors remain elusive; **Objective:** This study explores the potential link between distinctive personality profiles, particularly type D personality, and an increased risk of Long COVID; **Methods:** A retrospective cross-sectional study at Tel-Aviv Sourasky Medical Center’s Post-COVID clinic analyzed data from 373 Long COVID patients through comprehensive questionnaires covering Long COVID syndrome, Fibromyalgia criteria, personality assessments, social support, and subjective evaluations of cognitive decline, health and life quality. In total, 116 out of 373 patients completed the questionnaire, yielding a 31% participation rate; **Results:** Cluster analysis revealed two groups, with Cluster 1 (N = 58) exhibiting Type D personality traits while Cluster 2 (N = 56) not meeting criteria for Type D personality. In comparison to Cluster 2, Cluster 1 patients reported heightened anxiety, depression, reduced social support, increased pain symptoms, manifestations of fibromyalgia, cognitive decline, and poor sleep quality, contributing to a diminished quality-of-life perception; **Conclusions:** findings highlight diverse personality profiles among Long COVID patients, emphasizing the need for tailored care. This approach shows potential for improving Long COVID patient care, aligning with the evolving personalized medicine paradigm.

## 1. Introduction

The Coronavirus Disease 2019 (COVID-19) pandemic, caused by severe acute respiratory syndrome (SARS) coronavirus 2 (SARS-CoV-2), emerged in 2019 and rapidly unfolded into a worldwide pandemic that has continued waxing and waning over 3 years, manifesting as the most challenging global health crisis for over a century [1]. The COVID-19 pandemic has imposed far-reaching and heavy burdens on the general population’s physical health and, just as importantly, mental health, with people facing the hardships of quarantine, disease burden and anxiety during this time [2].

Most individuals diagnosed with COVID-19 typically exhibit symptoms such as fever, cough, dyspnea, and fatigue. However, the majority of these cases involve mild disease. Approximately 10–15% require hospitalization and oxygen supplementation, and this course is far more frequent among individuals with risk factors such as old age and comorbidities [3]. Typically, the majority of patients achieve full recovery within 3–4 weeks.

The COVID-19 pandemic has revealed a spectrum of neurological, neuropsychiatric, and psychological effects beyond its respiratory impact. Stressors such as social isolation, fear of infection, bereavement, financial strain, and unemployment have led to increased rates of depression, anxiety, adjustment disorders, insomnia, and substance abuse. Additionally, some patients experience persistent symptoms post-recovery, further aggravating these mental health issues. Individuals with pre-existing mental health conditions are particularly vulnerable. Addressing these mental health implications is crucial, emphasizing the need for accessible support systems and healthcare services [4].

Long COVID is a term used to describe the protracted illness presented by individuals who have recovered from the acute phase of COVID-19 but are still experiencing the usual clinical picture of COVID-19 for much longer than expected, or have new symptoms such as extreme fatigue, exercise intolerance, dyspnea, insomnia and “brain fog” [5]. Different criteria for Long COVID are used in research. The consensus usually entails suffering from one or more of the COVID-19 symptoms after a period of at least 1 month from the time of acute illness, with no alternative diagnosis [6].

Longer follow-up studies have demonstrated that the most frequent Long COVID-19 symptoms are fatigue (78%), post-exertional malaise (72%), and cognitive dysfunction (55%) [5]. Debilitating sequels with organ damage involving the pulmonary, cardiovascular, musculoskeletal and autonomic nervous systems have also been reported, though in much lower incidence [7]. This constellation of symptoms may overlap with those experienced by patients suffering from fibromyalgia (FM) and chronic fatigue syndrome (CFS) [8,9]. These symptoms can coincide and be exacerbated in patients already suffering from mental health disorders [10].

Equally significant are the extensive psychological sequels, including depressive symptoms and anxiety, reported by up to 35% of patients recovering from COVID-19. The psychopathological mechanisms driving post-COVID-19 depressive symptoms are primarily linked to inflammation triggered by the peripheral immune-inflammatory response to the viral infection and the enduring psychological burden during and after infection, including imposed isolation [11].

At first, this syndrome was more prevalent among patients with severe disease, patients of older age and those with multiple comorbidities, as well as among patients who were hospitalized and required oxygen supplementation or mechanical ventilation [12]. Over time, as the pandemic expanded, morbidity grew in the general population among young individuals and the elderly alike. Contrary to expectations, updated research has shown that the prevalence of Long COVID-19 was disproportionally high in young, non-hospitalized individuals with low disease burden [9,13]. 

Extensive research has been conducted to characterize individuals who are prone to developing Long COVID. A prospective cohort study assessing recovered patients found that the baseline clinical features associated with severe disease had no association with the risk of developing Long COVID [14]. Furthermore, studies have shown that Long COVID patients have no baseline clinical features associated with the development of the syndrome; yet surprising risk factors were demonstrated, including female sex and a young age between 35–50 years [15,16]. 

Several studies have focused on searching for common physical baseline characteristics for the development of this syndrome, yet no definitive results have been found thus raising the question of a baseline psychological common ground. First and foremost, a possible distinctive personality profile that encompasses specific psychological features could differentiate this population of non-hospitalized individuals with low disease burden. Notably, previous research has identified the so-called “type D personality” (“D” for distressed) as a risk factor for various physical outcomes, including prevalent conditions such as ischemic heart disease (IHD) [17,18].

A study conducted by Ablin et al. investigated personality types, using questionnaires, in patients with FM or CFS compared to controls, revealing significant differences in psychological variables between the two groups. FM/CFS patients showed a less adaptive pattern with a high prevalence of Type D personality [19,20]. These findings advocate for further research into the association between different personality profiles and specific syndromes and disorders. Our hypothesis posits that distinct patterns of coping mechanisms or traits could characterize individuals with Long COVID or render them more susceptible to the syndrome. In our current study, we aimed to provide a cross-sectional snapshot of patients diagnosed with Long COVID, assessing specific personality types, including Type D personality.

While our approach does not seek to establish a causal relationship between personality and clinical outcomes, it offers an important descriptive contribution to our understanding of Long COVID and may inform future research, including longitudinal studies.

## 2. Materials and Methods

### 2.1. Participants

A total of 373 participants were recruited from a pool of 750 individuals undergoing follow-up at the Tel Aviv Sourasky Medical Center post-COVID-19 clinic as outpatients. Their cases were meticulously examined. These patients were diagnosed with COVID-19, exhibiting asymptomatic to mild disease without the necessity for hospitalization or oxygen supplementation following the contraction of COVID-19. These individuals developed a prolonged illness, leading to a diagnosis of Long COVID Syndrome, as per prevailing definitions, characterized by the persistence of one or more symptoms for over a month: fatigue, cognitive decline, brain fog, dyspnea, or cough [6].

### 2.2. Procedure

All 750 individuals undergoing follow-up at the Long COVID clinic were diagnosed with Long COVID syndrome based on current clinical guidelines. All individuals were approached for participation via telephone call. In total, 373 were successfully contacted and were sent the study questionnaires via text message. Furthermore, 116 individuals completed the entire research questionnaire, reflecting a 31% completion rate. Two patients did not meet the Long COVID criteria of our study questionnaire and were consequently excluded from the research, leaving 114 participants. 

Eligibility criteria included meeting Long COVID diagnosis as mentioned above, mild course of COVID illness without hospitalization or oxygen supplementation, providing informed consent, and being above 18 years of age.

The study questionnaires included demographic details, background diseases, a questionnaire regarding symptoms and complaints, and several questionnaires regarding the participant’s personality characteristics, well-being, and mental health status. It was constructed using Qualtrics^XM^ program 2023, with secure access and an anonymous collection, with no possibility of connecting between replied questionnaires to participants.

### 2.3. Tools and Measures

The full format of the questionnaires is added in Supplementary Materials.

- The DS-14 questionnaire for assessing Type D personality (S1). A validated Hebrew version of the questionnaire by Zohar et al. was used [20]. Within our current sample, the internal consistency was α = 0.86.
- The Generalized Anxiety Disorder (GAD) questionnaire for assessing symptoms of anxiety, based on the Diagnostic and Statistical Manual of Mental Disorders-IV (S2). A validated Hebrew version of the questionnaire was used [21,22].
  The survey, known for its high internal reliability and validity (Löwe et al. (2008) [23]), exhibited robust internal consistency in the present sample (α = 0.95).
- The Patient Health Questionnaire (PHQ-9) for assessing the level of depression (S3). A validated Hebrew version of the questionnaire was used [24].
  In validation studies conducted by Kroenke et al. (2001) [25], the PHQ-9 demonstrated Cronbach’s alpha values of 0.89. Within our current sample, the internal consistency was solid (α = 0.84).
- The Multi-dimensional Perceived Social Support scale (MSPSS) for assessing a person’s subjective perception of the extent of his or her social support (S4). A validated Hebrew version of the questionnaire by Gross et al. was used [26]. Within our current sample, the internal consistency was robust (α = 0.92).
- The Pittsburgh Sleep Quality Index (PSQI) evaluates the quality and pattern of sleep self-reported by questionnaires (S5). A validated Hebrew version of the questionnaire was used [27]. The current version of the questionnaire, translated into Hebrew and validated by Shochat et al. (2007) [28], demonstrated solid internal consistency in our sample (α = 0.82).
- The subjective Cognitive Decline (SCD) questionnaire—assessing cognitive complaints by six questions regarding cognitive decline (S6). A validated Hebrew version of the questionnaire was used [29]. Developed by Elkana et al., this tool has exhibited correlations with objective cognitive abilities, overall subjective cognitive perception, and indices related to pain in individuals diagnosed with fibromyalgia. In the current sample, the internal consistency was robust (α = 0.95).
- The Widespread Pain Index (WPI)—calculated by documenting the number of sites where the patient has felt pain over the last week, out of a total of 19 specific-predesignated sites. Used predominantly in the diagnosis of FM (S7). A validated Hebrew version of the questionnaire was used [30].
- Symptom Severity Scale (SSS)—evaluates symptoms of fatigue, unrefreshing sleep, cognitive symptoms, and multiple related factors. Used predominantly in the diagnosis of FM (S8). A validated Hebrew version of the questionnaire was used [30] with solid internal consistency (α = 0.84).
- Life quality test SF-12 provides a subjective assessment of daily activity, physically and mentally (S9). A validated Hebrew version of the questionnaire was used [31] with solid internal consistency (α = 0.89).
- Long COVID questionnaire—The Long COVID questionnaire was crafted for the study, given the absence of a validated questionnaire at the time (S10). The design drew inspiration from similar surveys employed during the study period, aligning with the prevailing definitions of Long COVID at the time [32]. The questionnaire evaluates symptoms characteristic of Long COVID, such as respiratory symptoms, fatigue, and muscle pain, by their length and severity. Within our current sample, the internal consistency was solid (α = 0.88).

## 3. Results

### 3.1. Patient Characteristics

In total, 114 patients who met Long COVID Syndrome criteria agreed to participate in the study. Most participants, comprising 85 individuals (74.6%) were women, and their mean age was 44.5 years old. Most patients, numbering 78 (68.4%) were healthy prior to contracting COVID-19 and developing Long COVID syndrome. In total, 36 patients (31.6%) had known prior medical diseases: 9 suffered from asthma (24%), 9 suffered from fibromyalgia (24%), 5 from diabetes (13.8%), 6 from hypertension (16.6%) as shown in Table 1. None of the patients reported experiencing mental health issues, nor did they have previous psychiatric diagnoses. Moreover, 46 participants (40.3%) reported subjective limited function in daily activities (such as climbing stairs and attending household chores). 

### 3.2. Demographic Characteristics of Clusters

To evaluate patterns of psychological coping, cluster analysis was performed on 114 participants whose questionnaires were complete. The analysis yielded two clusters, Cluster 1 (N = 58) and Cluster 2 (N = 56). In total, 58 participants (50.8% of total patients) who suffered from Long COVID, met diagnostic criteria for type D personality, while 56 participants (49.12%) did not, respectively. The clusters differed from each other on psychological variables as type D personality diagnosis, yet did not differ regarding demographic characteristics including age, gender, background diseases, marital status, and physical status. Cluster 1, consisting of patients with Type D personality had a mean age of 44.9 (with SD 14.5). In total, 46 of the participants (79.3%) were females and 12 males (20.7%). Most patients were married (N = 32, 55.2%). Furthermore, 22 patients (38%) had previous chronic diseases as mentioned above and 27 patients (46.5%) suffered from daily function limitation.

## 4. Cluster Analysis

### Personality Traits of Clusters

In comparison to Cluster 2, Cluster 1 displayed a less adaptive pattern, increased levels of anxiety and depression, along with subjective perception of reduced levels of social support and a greater self-reported prevalence of cognitive decline. Additionally, Cluster 1 individuals with Long COVID experienced a higher burden of symptoms as manifested in a significantly higher total score. Matching our hypothesis, Cluster 1 patients suffered from a higher rate of neuropsychiatric complaints such as attention deficit, memory problems, insomnia, and headaches. Furthermore, this group reported more frequent pain manifestations, with a higher incidence of Fibromyalgia diagnosis based on WPI and SSS questionnaires. Consequently, overall, they perceived a lower quality of life as positivity reflected in the SF-12 questionnaire MCS—mental component score. Table 2 demonstrates significantly higher test scores in all fields for Cluster 1 consisting of individuals with Type D personality compared to Cluster 2.

Table 3 presents a comparison of the burden of anxiety, depression, poor sleep quality, and symptoms of diffuse pain interacting with Fibromyalgia (FM). It is evident that Cluster 1 is characterized by a significantly higher prevalence of complaints.

## 5. Discussion

The pathogenesis of Long COVID is multifactorial, encompassing various factors, including persistent infection, immune dysregulation, chronic inflammatory responses, and systemic dysfunction in both vascular and microvascular systems [33,34,35,36,37]. 

Recognizing the interaction between physical and psychological elements is pivotal to developing effective strategies for diagnosis, treatment, and long-term management of individuals grappling with the persistent manifestations of COVID-19.

The current study constitutes a novel exploratory approach towards characterizing Long COVID patients and identifying psychological patterns and personality traits of Long COVID patients. Using a cross-sectional approach, we attempted to identify personality traits prevalent among Long COVID patients, specifically focusing on the type D personality traits, previously associated with other complex chronic conditions. 

Individuals with Type D personality profiles are prone to negative affectivity, social inhibition and mental rigidity. Due to these negative cognitive patterns, these individuals tend to experience difficulty in acquiring social support, and thus tend to experience depression and feelings of loneliness and helplessness [38]. Additional difficulties encountered by such individuals include poor quality of sleep, anxiety, avoidance and isolation. Due to negative cognitive patterns, these individuals tend to experience their surroundings as critical, thus augmenting negative feelings [39].

In the current study, the Long COVID patients fulfilling Type D personality criteria exhibited notably elevated scores on the Long COVID questionnaire, indicating a greater burden of symptoms. These patients reported significantly higher frequencies of neuropsychiatric symptoms, including depression, memory loss, and attention span issues, as well as a heightened occurrence of somatic systemic symptoms in the cardiovascular, respiratory, and gastrointestinal domains. Moreover, patients with Type D personality appear to score significantly lower on measures associated with resilience such as positivity, feelings of social support, and overall quality of life, both physical and mental, reinforcing findings in previous studies [38,40,41]. These findings raise the possibility that Type D personality traits may act as risk factors for the development of Long COVID, although this hypothesis cannot be substantiated by the current cross-sectional study but rather would require a longitudinal approach [41,42,43].

Prior research has shown that having Type D personality traits makes individuals more vulnerable to other chronic pain disorders including Fibromyalgia (FM) and chronic fatigue syndrome [19,44]. Previous studies have identified Type D personality as a potential risk factor and prognostic marker for common illnesses, including ischemic heart disease (IHD), congestive heart failure (CHF), and arrhythmias [18,39,45]. Updated research has augmented these results, demonstrating the association between Type D personality and a lower quality of life in patients suffering from Atrial Fibrillation (AF) who underwent catheter ablation [45]. Additionally, in a larger systematic review, negative affectivity, a major trait of Type D personality, was associated with the incidence and clinical prognosis of AF [46].

The clinical and possible pathogenetic overlap between Long COVID and FM is an evolving concept that has attracted significant interest in the current literature [47,48]. 

FM, recently identified in terms of a nociplastic pain condition [49] has previously been associated with various infectious triggers and in this context, it is not surprising that COVID-19 has manifested itself as another such infectious trigger. On the other hand, the specific characteristics of Long COVID may help lead to a better understanding of mechanisms involved in the pathogenesis of FM. Similar to previous research [48], in the current study we were able to identify a high prevalence of FM symptoms among our Long COVID patients. Moreover, the high prevalence of Type D personality among our patients, as well as its clinical correlates, is in accordance with the previous findings regarding Type D personality among FM and CFS patients [20]. 

During this study, we explored the connection between developing Long COVID and other psychological traits such as those of Type D personality and individuals suffering from FM. In our study population, 37 patients with Long COVID met the diagnostic criteria for Fibromyalgia (FM) based on the WPI and SSS questionnaires. Moreover, 9 patients had a previous known diagnosis; however, for 28 patients (24.5%), it was a new diagnosis, emphasizing the association between the two syndromes.

Cluster 1 encompasses a significantly higher number of patients suffering from FM in comparison to Cluster 2 (PV < 0.001 for SSS and PV = 0.053 for WPI): 25 (43.1%) vs. 12 (21.4%), respectively, with a high rate (32.7% vs. 16%, respectively) of new diagnosis appearing simultaneously surrounding developing Long COVID syndrome. This observation strengthens the connection between Long COVID and FM, which is the focus of extensive research, indicating shared pathophysiology, risk factors, and similar manifestations [47]. 

Moreover, Cluster 1 showed a significantly higher score on the SSS questionnaire as seen in Table 2, indicating a higher burden of symptoms and severity of disease. On the one hand, the findings in this study serve to further strengthen the foundation of previous research, confirming the association between Type D personality and FM [50]. On the other hand, this prompts inquiries about the inherent connection between Fibromyalgia (FM) and Long COVID syndromes, suggesting that their symptoms may overlap on a spectrum and could be influenced by personality traits and coping mechanisms [19].

The outcomes of the present study carry significant implications for both therapy and further research. Emotional profiles, akin to those discovered in our study, have previously demonstrated associations with diverse facets of well-being and the ability to predict an individual’s future health status [51]. In prior studies, cooperativeness was strongly associated with perceived social support and, by proxy, with a sense of capability and independence, thereby contributing to an improvement in overall well-being. Meanwhile, self-transcendence exhibited a robust association with positive emotions and well-being, in stark contrast to the feelings of self-centeredness and pessimistic outlook that individuals with Type D personality traits tend to possess [52].

The group clustering outlined in the current study holds the potential for developing patient-specific treatment plans tailored to the needs of Long COVID patients, based on their coping style [53,54]. Early detection of individuals prone to developing Long COVID will enable prompt care, close monitoring, and follow-up, with the aim of cultivating resilience and self-confidence to minimize the long-standing sequelae of mental health issues and reduce the societal and economic burden of Long COVID syndrome. Furthermore, extensive research highlights the need for a multidisciplinary approach involving medical and psychological professionals to effectively mitigate the impact of this syndrome. Cognitive therapy is the frontline treatment, with SSRIs and Melatonin serving as important tools to reduce susceptibility to depression and manage associated comorbidities [55,56].

### Limitations

The study population was recruited from the Post COVID-19 clinic at Sourasky Medical Center after being diagnosed with Long COVID syndrome, based on prevailing definitions at the time. Consequently, there was no control group of individuals who contracted COVID-19 and did not develop Long COVID. Thus, the prevalence of Type D personality in healthy individuals post-COVID was not measured and relied on the general population prevalence, marked at 20%. Our data are cross-sectional and self-reported, making them susceptible to personal perceptual bias, as well as cultural and language bias. While the current study identified two distinct personality profiles within the Long COVID patient population, it is crucial to acknowledge that these profiles may not be strictly dichotomous. Instead, they may exist on a continuum, allowing patients to exhibit various degrees of alignment with either profile.

## 6. Conclusions

The study’s noteworthy findings indicate the presence of two distinct personality profiles among Long COVID syndrome patients. The clear clustering of patients into two almost equal groups, with a much higher rate of Type D personality diagnosis prevalence than in the general population, emphasizes the association between certain personality traits, such as Type D personality, and developing Long COVID. Furthermore, Long COVID patients with Type D personality showed a greater disease burden with lower quality of sleep, lower perception of quality of life, positivity, and social support. These results support and augment similar findings from previous studies, demonstrating that Type D individuals possess negative traits associated with increased risk for mental and physical disorders. 

These results carry the potential to raise awareness and facilitate early detection of such individuals, enabling prompt assessment and care to prevent, identify, and enhance the treatment of COVID-19 in individuals with Type D personality. A deeper comprehension of the connection between the Type D personality profile and Long COVID syndrome allows us to customize care to meet their specific requirements, psychological strengths, and vulnerabilities. The goal is to minimize the enduring consequences of mental health issues like anxiety and depression. This approach aligns with the current trend in personalized medicine, striving to address each patient’s unique needs and resources. 

However, there is still much unknown about Long COVID syndrome. Thus, further research is warranted to delve into the complete spectrum of personality traits among Long COVID patients. This exploration aims to achieve a more comprehensive understanding of the differences between these groups, potentially laying the groundwork for developing personalized and optimal treatments for individuals impacted by Long COVID to alleviate individual suffering and reduce the societal and economic burden of Long COVID syndrome.

## Figures and Tables

**Table 1:** Demographic details of study participants divided by clusters and in total.

		Cluster 1 (N = 58)	Cluster 2 (N = 56)	Total (N= 114)
Age		20–68	21–78	20–78
		mean: 43.49	mean: 45.96	mean: 44.5
		SD: 14.5	SD: 14.4	SD: 14.4
Sex	Male	12 (20.7%)	17 (30.4%)	29 (25.4%)
	Female	46 (79.3%)	39 (69.6%)	85 (74.5%)
Marital status	Married	32 (55.2%)	29 (51.8%)	61 (53.5%)
	Divorced	10 (17.2%)	12 (21.4%)	22 (19.2%)
	Widow	0	1 (1.8%)	1 (0.07%)
	Single	16 (27.6%)	14 (25%)	30 (26.3%)
Chronic disease		22 (37.9%)	14 (25.9%)	36 (31.5%)
	Asthma	3 (5.1%)	5 (8.9%)	8 (7%)
	Fibromyalgia	6 (10.3%)	3 (5.1%)	9 (7.8%)
	Diabetes	2 (3.4%)	3 (5.1%)	5 (4.3%)
	IBD\IBS	3 (5.2%)	2 (3.5%)	5 (4.3%)
	Hypertension	5 (8.6%)	1 (1.7%)	6 (5.2%)
	Hypothyroidism	4 (6.8%)	1 (1.7%)	5 (4.3%)
	Other	7 (10.7%)	7 (10.2%)	14 (12.2%)
Physical status	Limitation on daily Function	27 (46.5%)	19 (33.9%)	46 (40.3%)
	No limitation on daily function	31 (53.5%)	37 (66.1%)	68 (59.6%)

Abbreviations: Cluster 1—type D personality profile. Cluster 2—not meeting criteria for type D personality profile. IBS—irritable bowel syndrome; IBD—inflammatory bowel disease.

**Table 2:** Cluster analysis presenting mean values of psychological variables per cluster group.

	Cluster 1 Mean (sd)	Cluster 2 Mean (sd)	t(df) = [t-Value], *p* = [*p*-Value].
Long COVID	87.5 (31.65)	63.3 (34.01)	t(112) = −3.92, *p* < 0.001
SSS	8.17 (2.8)	5.78 (2.8)	t(112) = −4.49, *p* < 0.001
WPI	4.94 (4.7)	3.39 (3.7)	t(112) = −1.95, *p* = 0.053
PSQI	11.06 (5.1)	7.91 (4.4)	t(112) = −3.5, *p* = 0.001
GAD7	9.51 (6.5)	2.69 (2.2)	t(112) = −7.51, *p* < 0.001
PHQ9	13.03 (6.5)	6.44 (4.4)	t(112) = −6.28, *p* < 0.001
SCD	22.06 (10.1)	14.94 (10.4)	t(112) = −3.69, *p* < 0.001
MSPSS	30.22 (5.9)	32.57 (4.5)	t(112) = 2.38, *p* = 0.019
SF-12			
MCS	34.68 (10.7)	47.22 (10.1)	t(112) = 6.43, *p* < 0.001
PCS	38.78 (10.05)	40.86 (11.7)	t(112) = 1.006, *p* = 0.317

Abbreviations: SCD—subjective cognitive decline. GAD7—generalized anxiety disorder. PHQ9—Patient Health Questionnaire assessing depression. SSS—Symptom Severity Scale assessing FM. MSPSS—Multi-dimensional Perceived Social Support scale. WPI—The Widespread Pain Index. PSQI—The Pittsburgh Sleep Quality Index. SF-12—life quality test.

**Table 3:** Cluster analysis presenting psychological symptoms per cluster group.

		Cluster 1 (N = 58)	Cluster 2 (N = 56)	
Anxiety (GAD7)	Severe	16 (27.6%)	0	*p* < 0.001
	Moderate	8 (13.8%)	0	
	Mild	18 (31%)	11 (19.6%)	
	None	16 (27.6%)	45 (80.4%)	
Depression (PHQ9)	Severe	13 (22.4%)	3 (5.4%)	*p* < 0.001
	Moderate	14 (24.1%)	11 (19.6%)	
	Mild	15 (25.9%)	18 (32.1%)	
	None	5 (8.6%)	24 (42.9%)	
Sleep quality (PSQI)	Good	10 (17.2%)	17 (30.4%)	*p* = 0.1
	Disturbed	48 (82.8%)	39 (69.6%)	
FM (SSS, WPI)	Overall prevalence	25 (43.1%)	12 (21.4%)	*p* = 0.013
	New diagnosis	19 (32.7%)	9 (16.0%)	
	Prior diagnosis	6 (10.3%)	3 (5.3%)	

Abbreviations: GAD7—generalized anxiety disorder. PHQ9—Patient Health Questionnaire assessing depression. PSQI—The Pittsburgh Sleep Quality Index. SSS—Symptom Severity Scale assessing FM. WPI—The Widespread Pain Index.

## Data Availability

The data presented in this study are available on request from the corresponding author. The data are not publicly available in order to maintain participant privacy.

## Supplementary Materials

The following supporting information can be downloaded at: https://www.mdpi.com/article/10.3390/jcm13102886/s1.
