# Supplementary material for: Long COVID-19 Enigma: Unmasking the Role of Distinctive Personality Profiles as Risk Factors
Source: J Clin Med. 2024 May 14;13(10):2886. doi: 10.3390/jcm13102886 (PMC11122355; doi:10.3390/jcm13102886)
Supplement: Supplementary file 1 [file jcm-13-02886-s001.zip › jcm-2998281-supplementary.pdf]

Supplementary files

S1- DS-14 questionnaire (Hebrew validated version)

לפניך משפטים שבהם אנשים מתארים את עצמם. אנא קרא/י את המשפטים והקיף/פי בעיגול את התשובה המתאימה ביותר עבורך. אין תשובות נכונות או שגויות יש רק תאור הולם או לא הולם.

		לא נכון	יותר לא נכון מנכון	לא נכון ולא לא נכון	יותר נכון מלא נכון	נכון
1	אני יוצר קשר בקלות כשאני פוגש אנשים	0	1	2	3	4
2	אני מתרגש מדברים לא חשובים	0	1	2	3	4
3	אני מדבר עם זרים לעתים קרובות	0	1	2	3	4
4	אני מרגיש לא שמח לעתים קרובות	0	1	2	3	4
5	אני מתעצבן לעתים קרובות	0	1	2	3	4
6	אני נוטה להרגיש עצור במצבים חברתיים	0	1	2	3	4
7	אני רואה שחורות	0	1	2	3	4
8	קשה לי להתחיל שיחה	0	1	2	3	4
9	יש לי הרבה מצבי רוח רעים	0	1	2	3	4
10	אני אדם די סגור	0	1	2	3	4
11	אני מעדיף לשמור מרחק מאחרים	0	1	2	3	4
12	אני מודאג לגבי משהו הרבה מהזמן	0	1	2	3	4
13	הרבה פעמים יש לי מצב רוח מזופת	0	1	2	3	4
14	במצבים חברתיים קשה לי למצוא נושאים לשיחה	0	1	2	3	4

DS-14 questionnaire (English translation)

Below are sentences in which people describe themselves. Please read the sentences and circle the most appropriate answer for you. There are no correct or incorrect answers, just select the answer that fits you the most:

	NOT TRUE	MORE UNTRUE THAN TRUE	NOT TRUE AND NOT UNTRUE	MORE TRUE THAN UNTRUE	TRUE
I make contact easily when I meet people	0	1	2	3	4
I often make a fuss about unimportant things	0	1	2	3	4
I often talk to strangers	0	1	2	3	4
I often feel unhappy	0	1	2	3	4
I am often irritated	0	1	2	3	4
I often feel inhibited in social interactions	0	1	2	3	4
I take a gloomy view of things	0	1	2	3	4
I find it hard to start a conversation	0	1	2	3	4
I am often in a bad mood	0	1	2	3	4
I am a closed kind of person	0	1	2	3	4
I would rather keep other people at a distance	0	1	2	3	4
I often find myself worrying about something	0	1	2	3	4
I am often down in the dumps	0	1	2	3	4
When socializing, I don't find the right things to talk about	0	1	2	3	4

S2- Long Covid questionnaire

At the early stages of our research, Long Covid syndrome was a new phenomenon that had not been investigated, hence in contrast to other questionnaires presented in this study, there was no official questionnaire for diagnosis or validation of Long Covid. The following questionnaire was constructed based on similar surveys in studies addressing Long Covid cited in this work. An hebrew version of the questionnaire was sent in our study, for your convience we add an english version.

Long Covid questionnaire – English version

Please rank any symptom from 0 (no symptoms at all) to 10 (significant symptoms burden)

General symptoms:

Easily tired – 0 1 2 3 4 5 6 7 8 9 10

Muscle pain – 0 1 2 3 4 5 6 7 8 9 10

Loss of weight – 0 1 2 3 4 5 6 7 8 9 10

Respiratory symptoms

Shortness of breath – 0 1 2 3 4 5 6 7 8 9 10

Chest pain – 0 1 2 3 4 5 6 7 8 9 10

Palpitations – 0 1 2 3 4 5 6 7 8 9 10

Caugh – 0 1 2 3 4 5 6 7 8 9 10

Neuropsychological symptoms

Headaches – 0 1 2 3 4 5 6 7 8 9 10

Sleeping disturbences – 0 1 2 3 4 5 6 7 8 9 10

Hypersommelence – 0 1 2 3 4 5 6 7 8 9 10

Night terrors – 0 1 2 3 4 5 6 7 8 9 10

Brain fog and attention deficit – 0 1 2 3 4 5 6 7 8 9 10

Mood swings such as anxiety or depression – 0 1 2 3 4 5 6 7 8 9 10

Dermatological symptom

Loss of hair – 0 1 2 3 4 5 6 7 8 9 10

Rash – 0 1 2 3 4 5 6 7 8 9 10

Gastrointestinal symptoms

Abdominal pain – 0 1 2 3 4 5 6 7 8 9 10

Diarreah – 0 1 2 3 4 5 6 7 8 9 10

Constipation – 0 1 2 3 4 5 6 7 8 9 10

Have you been suffering from these symptoms for at least a month after being diagnosed with Covid-19? Yes\ No

Are you experiencing any other symptoms that werent mentioned above? If so, please eleborate ________

<u>Long Covid Questionnaire- Hebrew version</u>

אנא דרג/י כל סימפטום מ 0 (לא סובל כלל) עד 10 (סובל מהבעיה מאוד).

סימפטומים כלליים:

עייפות או התעייפות בקלות – 0 1 2 3 4 5 6 7 8 9 10

כאבי שרירים– 0 1 2 3 4 5 6 7 8 9 10

אובדן משקל– 0 1 2 3 4 5 6 7 8 9 10

סימפטומים נשימתיים וקרדיו-ווסקולרים:

קוצר נשימה – 0 1 2 3 4 5 6 7 8 9 10

כאב בחזה– 0 1 2 3 4 5 6 7 8 9 10

פלפיטציות (תחושת דפיקות לב) – 0 1 2 3 4 5 6 7 8 9 10

שיעול– 0 1 2 3 4 5 6 7 8 9 10

סימפטומים נוירופסיכיאטרים:

כאב ראש– 0 1 2 3 4 5 6 7 8 9 10

הפרעות שינה– 0 1 2 3 4 5 6 7 8 9 10

שינת-יתר– 0 1 2 3 4 5 6 7 8 9 10

סיוטי לילה– 0 1 2 3 4 5 6 7 8 9 10

קושי בזיכרון או בריכוז– 0 1 2 3 4 5 6 7 8 9 10

שינויי מצב רוח כגון דיכאון או חרדה– 0 1 2 3 4 5 6 7 8 9 10

סימפטומים עוריים:

אובדן שיער– 0 1 2 3 4 5 6 7 8 9 10

פריחה– 0 1 2 3 4 5 6 7 8 9 10

סימפטומי מערכת העיכול:

כאבי בטן– 0 1 2 3 4 5 6 7 8 9 10

שלשולים– 0 1 2 3 4 5 6 7 8 9 10

עצירות– 0 1 2 3 4 5 6 7 8 9 10

האם הסימפטומים נמשכים לפחות חודש מאז אובחנת כחולה ב COVID-19? כן / לא

האם את/ה סובל מסימפטומים נוספים אשר לא פורטו ברשימה לעיל? אם כן אנא פרט/י

S3- GAD 7 questionnaire (Hebrew validated version)

במהלך השבועיים האחרונים, באיזו תדירות היית מוטרד מכל אחת מן הבעיות הבאות?

(הקף בעיגול את תשובתך).

	כלל לא	בחלק מהימים	יותר ממחצית הימים	כמעט כל יום
הרגשתי עצבני, חרד או מתוח מאוד	0	1	2	3
לא הייתי מסוגל להפסיק לדאוג או לשלוט בדאגה	0	1	2	3
הייתי מודאג יותר מידי בנוגע לדברים שונים	0	1	2	3
התקשיתי להרגע	0	1	2	3
הייתי כל כך חסר מנוחה שהיה קשה לי לשבת בלי לזוז	0	1	2	3
הייתי מתעצבן או מתרגז בקלות	0	1	2	3
פחדתי כאילו משהו נורא עלול לקרות	0	1	2	3

GAD 7 questionnaire (English translation)

During the past two weeks, how often have you been bothered by each of the following problems:

	Not at all	Several days	More than half the days	Nearly every day
Feeling nervous, anxious, or on edge	0	1	2	3
Not being able to stop or control worrying	0	1	2	3
Worrying too much about different things	0	1	2	3
Trouble relaxing	0	1	2	3
Being so restless that it is hard to sit still	0	1	2	3
Becoming easily annoyed or irritable	0	1	2	3
Feeling afraid, as if something awful might happen	0	1	2	3

S4 - PHQ 9 questionnaire (Hebrew validated version)

במהלך השבועיים האחרונים, באיזו תדירות היית מוטרד מכל אחת מן הבעיות הבאות?

	כלל לא	במספר ימים	ביותר ממחצית מן הימים	כמעט כל יום
עניין מועט או הנאה מועטה מעשיית דברים	0	1	2	3
תחושת דכדוך, דכאון או חוסר תקווה	0	1	2	3
קשיים בהירדמות או שינה רציפה, או עודף שינה	0	1	2	3
תחושה של עייפות או אנרגיה מועטה	0	1	2	3
תיאבון מועט או אכילת יתר	0	1	2	3
מרגיש רע לגבי עצמך- מרגיש שאתה כישלון או שאכזבת את עצמך או את משפחתך	0	1	2	3
קושי להתרכז בדברים ,כמו קריאה בעיתון או צפייה בטלוויזיה	0	1	2	3
דיבור או התנהלות איטית עד כדי כך שאחרים הבחינו בכך, או להיפך- חוסר שקט ומנוחה, כך שהרגשת שאתה נע יותר מהרגיל	0	1	2	3
מחשבות על כך שעדיף שהייתי מת או מחשבות על פגיעה בעצמי בדרך כלשהי	0	1	2	3

במהלך השבועיים האחרונים, באיזו תדירות נתקלת בכל אחת מן הבעיות הבאות? (הקף בעיגול את תשובתך).

אם סימנת בעיות כלשהן, עד כמה בעיות אלו הקשו עליך לבצע את עבודתך, לטפל בדברים בבית או להסתדר עם אנשים אחרים?

☐ לא הקשו כלל

☐ הקשו במידת מה

☐ מאוד הקשו

☐ הקשו באופן קיצוני

PHQ 9 questionnaire (English translation)

During the past two weeks, how often have you been bothered by each of the following problems:

	Not at all	Several days	More than half the days	Nearly every day
Little interest or pleasure in doing things	0	1	2	3
Feeling down, depressed, or hopeless	0	1	2	3
Trouble falling or staying asleep, or sleeping too much	0	1	2	3
Feeling tired or having little energy	0	1	2	3
Poor appetite or overeating	0	1	2	3
Feeling bad about yourself — or that you are a failure or have let yourself or your family down	0	1	2	3
Trouble concentrating on things, such as reading the newspaper or watching television	0	1	2	3
Moving or speaking so slowly that other people could have noticed?<br>Or the opposite — being so fidgety or restless that you have been moving around a lot more than usual	0	1	2	3
Thoughts that you would be better off dead or of hurting yourself in some way	0	1	2	3

If you checked off any problems, how difficult have these problems made it for you to do yourwork, take care of things at home, or get along with other people?

- ☐ Not difficult at all.
- ☐ Somewhat difficult.
- ☐ Very difficult.
- ☐ Extremely difficult.

S5- MSPSS questionnaire (Hebrew validated version)

בשאלון זה אנו מבקשים לדעת מהי הרגשתך בנוגע למשפטים הבאים. השאלון מורכב מרשימת משפטים, אנא ענה/י את התשובה הנכונה ביותר עבורך. כאשר התשובות אינן נראות לך מתאימות או שקשה לך לענות על השאלה, גם אז חשוב שתבחר/י באחת מן האפשרויות שמתארת אותך בצורה נכונה יותר מהאחרות.

	**1**<br>**לא מסכים/ה כלל**	**2**<br>**מסכים/ה באופן חלקי**	**3**<br>**מסכים/ה**
יש אדם קרוב ללבי הנמצא בקרבתי כאשר אני נזקק/ת לכך.			
יש אדם קרוב ללבי אותו אני יכול/ה לשתף בצער ובשמחה.			
משפחתי באמת מנסה לעזור לי.			
אני מקבל/ת ממשפחתי את העזרה והתמיכה הרגשית לה אני זקוק/ה.			
יש אדם קרוב ללבי המהווה עבורי מקור עידוד ממשי.			
חברי באמת מנסים לעזור לי.			
אני יכול/ה לסמוך על חברי כאשר מתעוררות בעיות.			
אני יכול/ה לשוחח על בעיותיי עם משפחתי.			
יש חברים אותם אני יכול/ה לשתף בשמחתי ובצערי.			
יש אדם קרוב ללבי שרגשותיי חשובים לו.			
משפחתי מוכנה לעזור לי לקבל החלטות.			
אני יכול/ה לדבר על בעיותיי עם חברי.			

MSPSS questionnaire (English translation)

The below questionnaire consists of several statements. Please read each statement carefully and indicate how do you feel about each statement, by selecting the answer that fits you the most.

	**1**<br>**Disagree**	**2**<br>**Partially agree**	**3**<br>**Agree**
There is a special person who is around when I am in need			

There is a special person with whom I can share joys and sorrows			
My family really tries to help me			
I get the emotional help & support I need from my family			
I have a special person who is a real source of comfort to me			
My friends really try to help me			
I can count on my friends when things go wrong			
I can talk about my problems with my family			
have friends with whom I can share my joys and sorrows			
There is a special person in my life who cares about my feelings			
My family is willing to help me make decisions			
I can talk about my problems with my friends			

S6- PSQI questionnaire (Hebrew validated version)

השאלות הבאות מתייחסות לדפוסי השינה שלך בחודש האחרון בלבד. בתשובותיך התייחס/י באופן המדויק ביותר לדפוס או ההתנהגות המייצגים באופן הטוב ביותר את מרבית הימים והלילות בחודש האחרון. אנא ענה/י על כל השאלות.

בחודש האחרון:

1. באיזו שעה את/ה הולך/ת לישון בד"כ? ______________
2. כמה זמן לוקח לך להירדם בד"כ? (מרגע כיבוי האור) ______________
3. באיזו שעה את/ה קם/ה בד"כ ? ______________
4. כמה שעות שינה את/ה נטו ישן/ה בד"כ ? (לא לכלול זמן שאת/ה ער/ה במטה, רק שעות שינה) ______________

5. בחודש האחרון, באיזו תדירות התקשית לישון מהסיבות הבאות	[0] לא בחודש האחרון	[1] פחות מפעם בשבוע	[2] פעם פעמיים בשבוע	[3] שלוש פעמים או יותר בשבוע
א. לקח 30 דקות או יותר להירדם				
ב. התעורר/ת בלילה או מוקדם לפנות בוקר				
ג. קמת לשירותים				
ד. התקשית לנשום				

ה. השתעלת או נחרת				
ו. קר מדי				
ז. חם מדי				
ח. חלומות בלהה				
ט. סובלת מכאבים				
י. סיבה אחרת (פרט): __________________ ________________________________				
6. בחודש האחרון, כמה פעמים לקחת תרופה (במרשם או ללא מרשם) לעזור לך לישון?				
7. בחודש האחרון, כמה פעמים התקשית להישאר ער/ה בזמן נהיגה, אכילה, שיחה עם חברים?				
8. בחודש האחרון, עד כמה התקשית להרגיש התלהבות מספקת כדי לבצע מטלות?				
9. בחודש האחרון, כיצד היית מדרג/ת את השינה שלך?	[0] מצוין	[1] טוב	[2] לא טוב	[3] גרוע

PSQI questionnaire (English translation)

The following questions refer to your sleep patterns in the last month only. In your answers, please refer as accurately as possible to the pattern or behavior that best represents most days and nights in the past month. Please answer all questions:

1. At what time do you usually go to sleep? ______________
2. How long does it usually take you to fall asleep? (From the moment the light is turned off) ______________
3. At what time do you usually wake up? ______________
4. How many hours of actual sleep do you usually get? (Not including time spent awake in bed, only sleeping hours) ______________

	[0] Not in the last month	[1] Less than once a week	[2] 1-2 times a week	[3] 3 or more times a week
5. In the past month, how often did you have difficulty sleeping due to the following reasons?				
a. It took 30 minutes or longer to fall asleep				
b. Waking up during the night or early in the morning				
c. Getting up to use the bathroom				

d. Difficulty breathing				
e. Coughing or snoring				
f. Feeling too cold				
g. Feeling too warm				
h. Vivid dreams				
i. Feeling pain				
j. Other reason (please elaborate): ________________ ________________				
6. In the last month, how many times did you take medication (prescription or over-the-counter) to help you sleep?				
7. In the last month, how many times did you have difficulty staying awake during activities such as driving, eating, or talking to friends?				
8. In the last month, how often did you have difficulty feeling enthusiastic enough to carry out tasks?				
9. In the last month, how would you rate your sleep?	[0] Great	[1] Good	[2] Not good	[3] Bad

S7- SCD questionnaire (Hebrew validated version)

1. דרג/י בבקשה עד כמה את/ה מרגיש/ה שאת/ה מתקשה לשלוף שמות של אנשים בשיחה פתוחה.
0 (ללא בעיות)-3 (חומרה קשה)
עד כמה לתחושתך החמירה התופעה ביחס לעבר. 0 (ללא שינוי) 3 במידה רבה

2. דרג/י בבקשה עד כמה את/ה מרגיש/ה שײַחוט המחשבה שלך נקטע" באמצע שיחה. 0 (ללא בעיות)-3 (חומרה קשה)
עד כמה לתחושתך החמירה התופעה ביחס לעבר. 0 (ללא שינוי) 3 במידה רבה

3. דרג/י בבקשה עד כמה את/ה מרגיש/ה שאינך זוכר/ת מטרה שלשמה הגעת לאחד מחדרי הבית או דבר שרצית לעשות ושכחת.
0 (ללא בעיות)-3 (חומרה קשה)
עד כמה לתחושתך החמירה התופעה ביחס לעבר. 0 (ללא שינוי) 3 במידה רבה

4. דרג/י בבקשה עד כמה את/ה מרגיש/ה שפעולות יומיומיות לוקחות יותר זמן. 0 (ללא בעיות)-3 (חומרה קשה)
עד כמה לתחושתך החמירה התופעה ביחס לעבר. 0 (ללא שינוי) 3 במידה רבה

5. דרג/י עד כמה את/ה נמנע/ת מפעולות מורכבות כגון פעולות רב שלביות, קבלת החלטות, תכנון מורכב של פעולות.
0 (ללא בעיות)-3 (חומרה קשה)
עד כמה לתחושתך החמירה התופעה ביחס לעבר. 0 (ללא שינוי) 3 במידה רבה

6. דרג/י בבקשה עד כמה את/ה מרגיש/ה ירידה בתפקודי החשיבה שלך (שליפת מידע, קשב, זיכרון) 0 (ללא בעיות)-3 (חומרה קשה)
עד כמה לתחושתך החמירה התופעה ביחס לעבר. 0 (ללא שינוי) 3 במידה רבה

SCD questionnaire (English translation)

The questionnaire consists of a list of statements and questions. Please choose the most appropriate answer for you. If the options do not seem suitable to you or if it is difficult for you to answer the question, it is still important to select one of the options that describes you more accurately than the others.

1. A. Please rate how often you find it difficult to recall the names of people you have met previously.

Rarely	Sometimes	Often	Almost Always
0	1	2	3

B. To what extent do you feel this has changed compared to the past?

No Change	Slight Change	Moderate Change	Significant Change

0	1	2	3

2. A. Please rate how often you feel you lose your "train of thought".

Rarely	Sometimes	Often	Almost Always
0	1	2	3

B. To what extent do you feel this has changed compared to the past?

No Change	Slight Change	Moderate Change	Significant Change
0	1	2	3

3. A. Please rate how often you forget why you entered a room or what you were going to do.

Rarely	Sometimes	Often	Almost Always
0	1	2	3

B. To what extent do you feel this has changed compared to the past?

No Change	Slight Change	Moderate Change	Significant Change
0	1	2	3

4. A. Please rate how difficult and time-consuming you find carrying out daily activities:

Not at all	Slightly	Moderately	Very Much
0	1	2	3

B. To what extent do you feel this has changed compared to the past?

No Change	Slight Change	Moderate Change	Significant Change
0	1	2	3

5. A. Please rate how often you avoid making decisions or planning activities.

Rarely	Sometimes	Often	Almost Always
0	1	2	3

B. To what extent do you feel this has changed compared to the past?

No Change	Slight Change	Moderate Change	Significant Change
0	1	2	3

6. A. Please rate to what extent you feel there has been a decline in your ability to think / remember / concentrate.

Not at all	Slightly	Moderately	Very Much
0	1	2	3

B. To what extent do you feel this has changed compared to the past?

No Change	Slight Change	Moderate Change	Significant Change
0	1	2	3

Subjective Cognitive Decline (SCD-6) Questionnaire: A self-report questionnaire, comprising six items, assesses the extent of cognitive difficulties experienced by participants. The scale ranges from 0 (rarely/not at all) to 3 (almost always/ significant change). Each item prompts participants to evaluate how specific cognitive aspects have declined compared to their previous experiences, using

a scale from 0 (no change) to 3 (significant change). Scores range from 0 to 36, with higher scores indicating a more pronounced and severe subjective cognitive decline.

S8- Widespread pain index (WPI) questionnaire (Hebrew validated version)

סמן באילו מהאזורים הבאים הרגשת כאב במהלך **השבוע האחרון**:

כתף שמאל	שוק שמאל
כתף ימין	שוק ימין
זרוע עליונה שמאל	לסת שמאל
זרוע עליונה ימין	לסת ימין
אמה שמאל	חזה
אמה ימין	בטן
עכוז שמאל	צוואר
עכוז ימין	גב עליון
ירך שמאל	גב תחתון
ירך ימין	אף לא אחד מאזורים אלו

- יש לספור את מספר האזורים שסומנו על מנת לקבל את אינדקס הכאב המפושט **(WPI)**_________

Widespread pain index (WPI) questionnaire (English translation)

Please check the areas in which you have experienced pain over the past week:

Left shoulder	Left lower leg
Right shoulder	Right lower leg

☐	Left upper arm	☐	Left jaw
☐	Right upper arm	☐	Right jaw
☐	Left lower arm	☐	Chest
☐	Right lower arm	☐	Abdomen
☐	Hip buttock left	☐	Neck
☐	Hip buttock right	☐	Upper back
☐	Upper leg left	☐	Lower back
☐	Upper leg right	☐	None of the areas

Count the number of areas marked to obtain the widespread pain index **WPI**________

S9- Symptom severity scale (SSS) questionnaire (Hebrew validated version)

א. יש לציין את חומרת התסמינים הבאים במהלך השבוע האחרון תוך שימוש בסקאלה הבאה:

**עייפות**	**יקיצה בלתי רעננה**	**תסמינים קוגנטיביים (ריכוז וזיכרון)**
0 - ללא בעיות	0- ללא בעיות	0 - ללא בעיות
1- חומרה קלה	1- חומרה קלה	1 - חומרה קלה
2- חומרה בינונית	2- חומרה בינונית	2 - חומרה בינונית
3 - חומרה קשה	3 - חומרה קשה	3 - חומרה קשה

יש לסכם את התוצאה של חלק זה ולרשום אותה כאן:__________(0-9)

ב. איזה מהתסמינים הבאים היו קיימים במהלך 6 החודשים האחרונים?

☐ כאבי ראש

☐ כאב/עוויתות בבטן התחתונה

דיכאון

יש להוסיף את מספר התסמינים הללו לתוצאה של חלק א' כדי לקבל את מדד חומרת התסמינים (0-12)

SSS____ציון_ סופי:

Symptom severity scale (SSS) questionnaire (English translation)

A. Please indicate the severity of the following symptoms over the past week using the following scale:

**Fatigue**	**Waking unrefreshed**	**Cognitive symptoms**
0 – no problems	0 – no problems	0 – no problems
1 – slight or mild problems	1 – slight or mild problems	1 – slight or mild problems
2 – moderate; considerable problems	2 – moderate; considerable problems	2 – moderate; considerable problems
3 – severe; pervasive; life disturbing problems	3 – severe; pervasive; life disturbing problems	3 – severe; pervasive; life disturbing problems

Please sum the results of this section and write it here: __________(0-9)

B. Which of the following symptoms have been present during the past 6 months?

Headaches

Abdominal pain

Depression

Add the number of these symptoms to the result of Part A to get the symptom severity score (0-12)

SSS final score: ______

S10- life quality SF12 (12 item short form survey) questionnaire (Hebrew validated version)

א. כיצד היית מעריך/ה את מצב בריאותך באופן כללי?

1. מצוין 2. טוב מאד 3. טוב 4. לא כל כך טוב 5. גרוע.

השאלות הבאות מתייחסות לפעילות שאת/ה עשוי/ה לבצע במשך יום רגיל.

<u>באיזו מידה מצב בריאותך מגביל אותך כעת ביצוע הפעולות הבאות:</u>

ב. פעילויות כמו הזזת שולחן, שימוש בשואב אבק, פעילות ספורטיבית מתונה כמו שחיה.

1. מגביל מאד 2. מגביל מעט 3. לא מגביל כלל

ג. עליה של מספר קומות במדרגות

1. מגביל מאד 2. מגביל מעט 3. לא מגביל כלל

בארבעת השבועות האחרונים כתוצאה ממצב בריאותך הגופני, האם היו לך אחת או יותר מהבעיות הבאות במסגרת עבודתך או בפעילויות יומיומיות רגילות אחרות.

ד. האם הפחתת את כמות הזמן שהקדשת לעבודתך או לפעילויות אחרות?

1. כן 2. לא

ה. האם היית מוגבל/ת בביצוע סוג כלשהו של עבודה או פעילויות אחרות?

1. כן 2. לא

בארבעת השבועות האחרונים, האם היו לך אחת או יותר מהבעיות הבאות במסגרת עבודתך או בפעילויות יומיומיות רגילות אחרות כתוצאה מבעיות רגשיות כלשהן (למשל הרגשת מדוכא/ה או חרד/ה?)

ו. האם הפחת את כמות הזמן שהקדשת לעבודתך או לפעילויות אחרות?

1. כן 2. לא

ז. האם לא עשית את עבודתך או פעילויות אחרות במידת ההקפדה הרגילה?

1. כן 2. לא

ח. **בארבעת השבועות האחרונים, עד כמה הפריעו כאבים לעבודתך הרגילה (כולל עבודה מחוץ לבית ועבודות בית)?**

1. כלל לא 2. מעט 3. במידה מתונה (ככה ככה) 4. די הרבה 5. הרבה מאד

<u>השאלות הבאות מתייחסות להרגשתך ולאופן בו הסתדרו לך דברים בארבעת השבועות האחרונים. עבור כל שאלה אנא סמן/י תשובה אחת שקרובה ביותר להרגשתך.</u>

כמה מהזמן במשך ארבעת השבועות האחרונים:

ט. הרגשת שלוו/ה ורגוע/ה?

1. כל הזמן 2 . רוב הזמן 3 . חלק ניכר מהזמן 4 . חלק מהזמן 5 . מעט מהזמן 6. אף פעם לא

י . היית מלא/ אנרגיה?

1. כל הזמן 2 . רוב הזמן 3 . חלק ניכר מהזמן 4 . חלק מהזמן 5 . מעט מהזמן 6. אף פעם לא

יא. הרגשת שחוק/ה או סחוט/ה?

1. כל הזמן 2 . רוב הזמן 3 . חלק ניכר מהזמן 4 . חלק מהזמן 5 . מעט מהזמן 6. אף פעם לא

יב . בארבעת השבועות האחרונים עד כמה הפריעו מצב בריאותך הגופני או בעיותיך הרגשיות לפעילותיך החברתיות (כמו: ביקור אצל חברים או קרובי משפחה)?

1. כל הזמן 2. רוב הזמן 3. חלק מהזמן 4. מעט מהזמן 5. כלל לא

Life quality SF12 questionnaire (English translation)

1. **In general, would you say your health is**:

1. Excellent. 2. Very good. 3. Good. 4. Fair. 5. Poor.

The following questions are about activities you might do during a typical day.

Does <u>your health now limit you</u> in these activities? If so, how much?

2. **Moderate activities** such as moving a table, using a vacuum cleaner:

1. Very limited. 2. A bit limited. 3. Not limited at all.

3. Climbing **several** flights of stairs:

1. Very limited. 2. A bit limited. 3. Not limited at all.

During the <u>past 4 weeks</u>, have you had any of the following problems with your work or other regular daily activities <u>as a result of your physical health</u>?

4. **Accomplished less** than you would like.

a. Yes. b. No.

5. Was limited in the **kind** of work or other activities.

a. Yes. b. No.

During the <u>past 4 weeks</u>, have you had any of the following problems with your work or other regular daily activities <u>as a result of any emotional problems</u> (such as feeling depressed or anxious)?

6. Accomplished less than you would like.

a. Yes. b. No.

7. Did work or activities **less carefully than usual**.

a. Yes. b. No.

8. **During the past 4 weeks, how much did pain interfere with your normal work (including work outside the home and housework)?**

a. Not at all. b. A little bit. c. Moderately. d. Quite a bit. e. Extremely.

These questions are about how you have been feeling during the past 4 weeks.
For each question, please provide the answer that best describes the way you have been feeling.

How much of the time during the past 4 weeks…

9. Have you felt calm & peaceful?

a. All the time. b. Most of the time. c. A good bit of the time. d. Some of the time.
e. A little of the time. f. None of the time.

10. Did you have a lot of energy?

a. All the time. b. Most of the time. c. A good bit of the time. d. Some of the time.
e. A little of the time. f. None of the time.

11. Have you felt downhearted and blue?

a. All the time. b. Most of the time. c. A good bit of the time. d. Some of the time.
e. A little of the time. f. None of the time.

12. **During the past 4 weeks, how much of the time has your physical health or emotional problems interfered with your social activities (like visiting friends, relatives, etc.)?**

a. All the time. b. Most of the time. c. Some of the time. d. A little of the time.
e. None of the time.
